# Neutrophils Do Not Express IL-17A in the Context of Acute Oropharyngeal Candidiasis

**DOI:** 10.3390/pathogens4030559

**Published:** 2015-07-24

**Authors:** Anna R. Huppler, Akash H. Verma, Heather R. Conti, Sarah L. Gaffen

**Affiliations:** 1 Children’s Hospital of Pittsburgh of UPMC, Department of Pediatrics, Pittsburgh, PA 15224, USA; E-Mail: ahuppler@mcw.edu; 2 Medical College of Wisconsin, Department of Pediatrics, Division of Infectious Diseases, Milwaukee, WI 53201, USA; 3 Department of Medicine, Division of Rheumatology & Clinical Immunology, University of Pittsburgh, BST S702, 200 Lothrop Street, Pittsburgh, PA 15261, USA; E-Mails: akash.verma@pitt.edu (A.H.V.); hrc11@pitt.edu (H.R.C.)

**Keywords:** IL-17, Th17, *Candida albicans*, neutrophils, fungal infection, oral candidiasis, innate lymphocyte, TCR, γδ-T cell, mice

## Abstract

IL-17 protects against pathogens by acting on nonhematopoietic cells to induce neutrophil recruitment through upregulation of chemokines and G-CSF. IL-17- and Th17-deficient humans and mice are susceptible to mucosal *Candida albicans* infections*,* linked to impaired neutrophil responses. IL-17 production is traditionally associated with CD4^+^ Th17 cells. However, IL-17 is also expressed during innate responses to facilitate rapid pathogen clearance. Innate IL-17-expressing cells include various lymphocyte-type subsets, including ILC3, NKT, γδ-T and “natural” Th17 (nTh17) cells. Some reports suggest that neutrophils can express IL-17 during fungal infections. Here, we asked whether neutrophils serve as a source of IL-17 during acute oropharyngeal candidiasis (OPC) using an IL-17A fate-tracking reporter mouse. Mice were subjected to OPC for two days, and oral tissue was analyzed by flow cytometry. IL-17A was expressed by γδ-T cells and TCRβ^+^ natural Th17 (nTh17) cells, as recently reported. Although infiltrating neutrophils were recruited to the tongue following infection, they did not express the IL-17A reporter. Moreover, neutrophil-depleted mice exhibited normal transcription of both *Il17a* and downstream IL-17-dependent gene targets after *Candida* challenge. Thus, in acute OPC, neutrophils are not a measurable source of IL-17 production, nor are they necessary to trigger IL-17-dependent gene expression, although they are essential for ultimate pathogen control.

## 1. Introduction

*Candida albicans* is a commensal fungus that colonizes the human oral cavity, skin and other mucosal tissues. In settings of immune compromise, such as HIV/AIDS, chemotherapy, organ transplantation or congenital immunodeficiency, *C. albicans* causes opportunistic infections, including oropharyngeal candidiasis (OPC, thrush) and chronic mucocutaneous candidiasis (CMC), which encompasses OPC, as well as infections of skin, nails and vaginal tract [1]. Recently, it has become clear that IL-17 (also known as IL-17A), the signature cytokine of the Th17 lineage, is critically important for immunity against *C. albicans* [2,3].

IL-17 is a pro-inflammatory cytokine that is a major driver of neutrophil responses *in vivo*. IL-17 acts predominantly on non-hematopoietic cells, such as epithelial cells, keratinocytes and mesenchymal cells, which express the heterodimeric IL-17 receptor composed of IL-17RA and IL-17RC [4]. Stimulation of these cells by IL-17 induces the expression of factors that amplify and recruit neutrophils to sites of infection, including G-CSF, CXC chemokines (IL-8, CXCL1, CXCL2, CXCL5) and S100A8/9 (calprotectin). Neutrophils are vital for immunity to OPC, as their depletion leads to dramatically-increased susceptibility to disease, and IL-17 deficiency is associated with reduced neutrophil mobilization [4,5]. Therefore, the IL-17-neutrophil axis is a central feature of immunity to mucosal *Candida* infections.

Immunity to OPC is traditionally associated with CD4^+^ T cells, particularly since over 90% of HIV/AIDS patients experience episodes of thrush during the course of disease [6,7]. Similarly, mice with an HIV transgene are susceptible to OPC, which is dependent on Th17-dervied cytokines [8]. T cells with reactivity to *C. albicans* in humans and mice are mainly of the Th17 phenotype [9,10,11,12]. Humans and mice lacking IL-17R signaling are subject to CMC, and mutations that impact Th17 cell generation or maintenance also predispose to *Candida* infections [4,13,14,15,16]. Unlike humans, mice are immunologically naive to *C. albicans,* as they do not harbor this organism as a commensal microbe [17]. Therefore, mice do not generate adaptive Th17 responses until at least 7–14 days post exposure to *Candida* [11]. Nonetheless, mice mount a rapid and effective IL-17-dependent innate response to oral *Candida* infection [4]. Therefore, both adaptive and innate immunity to OPC is mediated by the IL-17R pathway.

In addition to conventional Th17 cells, numerous innate cell types express IL-17 [18]. Such cells can be mobilized rapidly in response to infection and do not require prior antigen exposure to be activated. In the context of both oral and dermal candidiasis, γδ-T cells have been shown to produce IL-17 [19,20,21]. In addition, we recently showed that a CD4^+^ innate lymphocyte population, known as “natural” Th17 (nTh17) cells, is also a major source of IL-17 in OPC [19]. Several recent reports have suggested that neutrophils may be an important source of IL-17 in the context of infections caused by fungal species, such as *Aspergillus* and *Fusarium* [22,23,24,25]. Since both IL-17 and neutrophils are essential for immunity to mucosal candidiasis, we asked whether this might also be the case in oral candidiasis. Using a sensitive IL-17 reporter mouse system, we find that neutrophils are not an apparent source of IL-17, nor are neutrophils required for induction of an IL-17 gene signature in the context of OPC.

## 2. Results

### 2.1. Neutrophils Do Not Produce IL-17 in Response to Oral C. albicans Infection

To determine whether neutrophils could be a source of IL-17 during OPC, we took advantage of an elegant fate-tracking reporter mouse that permits sensitive detection of IL-17A in oral immune cells [20]. In this system, the CRE recombinase is expressed under the control of the endogenous *Il17a* promoter and crossed to a Rosa26eYFPflox/stop mouse line (Il17a^Cre^Rosa26R^eYFP^, referred to as IL-17^eYFP^). Consequently, cells that express the *Il17a* gene become permanently marked by YFP and, therefore, report both prior and current IL-17 expression. Importantly, mice heterozygous for CRE are not deficient for IL-17 due to retention of IL-17A on one allele, and, therefore, show normal resistance to OPC [26]. This system was used originally to demonstrate that IL-17A is produced by γδ-T cells in acute dermal candidiasis [20]. We recently used these mice to demonstrate that IL-17A is produced by γδ-T and nTh17 cells during the early (two day) response to oral candidiasis [19].

**Figure 1:**
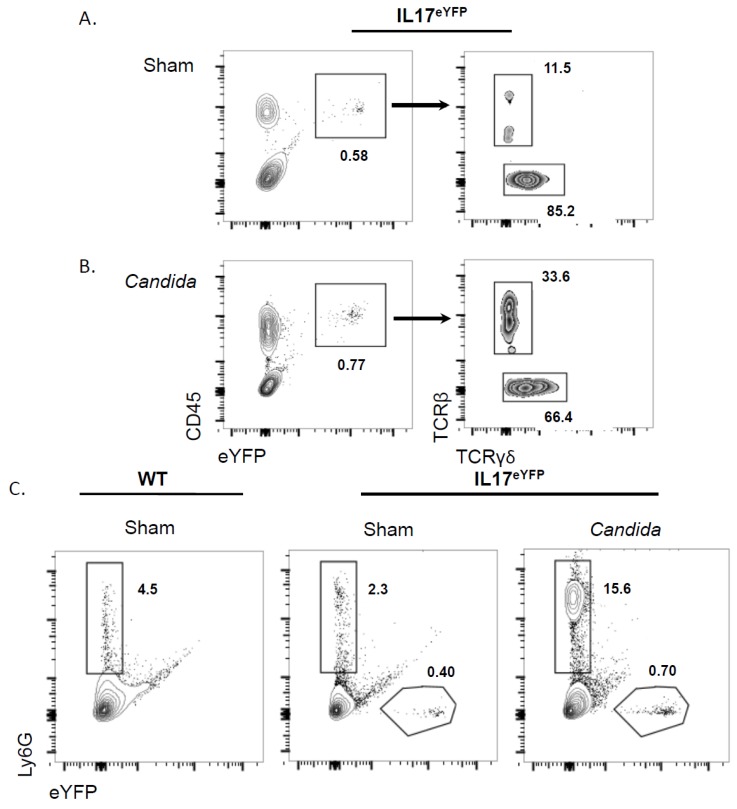
TCR^+^ cells, but not neutrophils, express IL-17A during acute oropharyngeal candidiasis (OPC). IL-17^eYFP^ mice were sham-infected (**A**) or orally challenged with *C. albicans* for 48 h (**B**). Single-cell suspensions from tongues (*n =* 5) were pooled and stained for CD45, TCRβ and TCRγδ. Left panels depict CD45^+^ and YFP^+^ cells within the lymphocyte gate. Right panels depict TCRγδ^+^ and TCRβ^+^ cells within the CD45^+^YFP^+^ gate. Percentages of populations are indicated. Data are representative of two independent experiments. (**C**) WT or IL-17^eYFP^ mice were sham infected or orally challenged with *C. albicans* for 48 h. Single-cell suspensions from tongues (*n =* 5) were pooled and stained for Ly6G. Panels depict Ly6G^+^YFP^+^ cells within the granulocyte gate. Percentages of populations are indicated. Data are representative of two independent experiments.

To determine whether neutrophils might also produce IL-17 during OPC, we employed a standard model system in which C57BL/6 (wild-type, WT) or IL-17^eYFP^ mice were infected orally by a 75-min sublingual exposure of *Candida albicans* [27]. Tongue tissue was collected two days post-infection and processed for flow cytometry. In sham-infected IL-17^eYFP^ mice, there was a small baseline population of CD45^+^YFP^+^ cells, indicating present or prior IL-17 expression (Figure 1A). These YFP^+^ cells were all TCR^+^, and the majority (85%) were TCR γδ^+^. After oral *C. albicans* infection, the CD45^+^YFP^+^ population reproducibly increased, and the percentage of TCRβ^+^ cells increased relative to the TCR γδ^+^ population (Figure 1B). The absolute number of YFP^+^ cells approximately doubled (data not shown). As expected, there was no detectable YFP signal in control WT mice (data not shown; [19]). These results are highly concordant with our recent observations and confirm that both TCRβ-expressing and TCR γδ-expressing cells contribute to the IL-17-dependent response to OPC [19].

To determine whether any IL-17 is expressed in neutrophils during acute OPC (two days post-infection), parallel samples of tongue homogenates were stained with Abs against Ly6G. In WT control mice, there was no baseline YFP^+^ population, as expected (Figure 1C). A population of Ly-6G^+^ cells representing neutrophils was present at low levels at baseline (2%–5%) in Sham-treated WT and IL17^eYFP^ mice. This Ly6G^+^ population expanded dramatically (to 15.6%) in *Candida-*inoculated IL-17^eYFP^ mice (Figure 1C). However, no cells in this population were positive for YFP, indicating that there was no apparent current or prior production of IL-17A by this cell population. The YFP^+^ population expanded (from 0.4%–0.7%), consistent with Figure 1A,B. Thus, there is no apparent production of IL-17A by neutrophils during the acute response to oral candidiasis.

### 2.2. Neutrophil Depletion Is Not Required for IL-17 Production or Signaling in OPC

**Figure 2:**
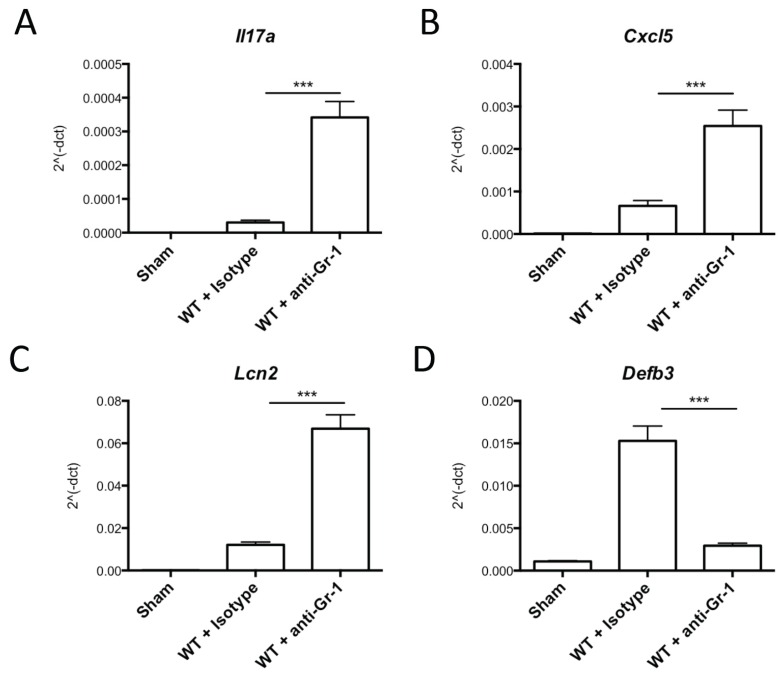
Neutrophil depletion does not impair expression of IL-17A or IL-17A-dependent signaling. Mice were injected intraperitoneally (i.p.) with anti-Gr-1 Ab, isotype Ab or PBS 24 h prior to inoculation with *C. albicans* or sham-infection. RNA was prepared from tongue homogenates 24 h after inoculation and subjected to qPCR for the indicated genes in triplicate: (**A**) *Il17a*; (**B**) *Cxcl5*; (**C**) *Lcn2*; and (**D**) *Defb3*. *** *p <* 0.05 by Student’s *t*-test compared to isotype-treated control mice.

As an independent approach to determine whether neutrophils contribute to IL-17 production, we depleted neutrophils in mice with anti-Gr-1 monoclonal Abs injected 24 h prior to infection, a regimen that we previously showed causes effective neutrophil depletion in tongue and renders mice susceptible to OPC [5,28]. We then assessed levels of IL-17A mRNA and expression of known IL-17 target genes following *C. albicans* infection. Control mice were treated with an isotype-matched Ab or sterile PBS. One day after administration, mice were inoculated orally with *C. albicans,* and gene expression in tongue was assessed by qPCR 24 h later. WT mice infected with *C. albicans* showed increased expression of *Il17a* mRNA*,* as expected (Figure 2A). *Il17a* expression was also enhanced in mice depleted of neutrophils; in fact, *Il17a* expression was markedly increased in infected mice depleted of neutrophils compared to controls (Figure 2A). This increase in *Il17a* may result from the failure of these mice to clear *C. albicans*, which is associated with enhanced inflammation, even at early time points when fungal loads are similar, and is consistent with previous studies [29]. This finding supports the conclusion that neutrophils do not express IL-17A during the early response to OPC.

**Figure 3:**
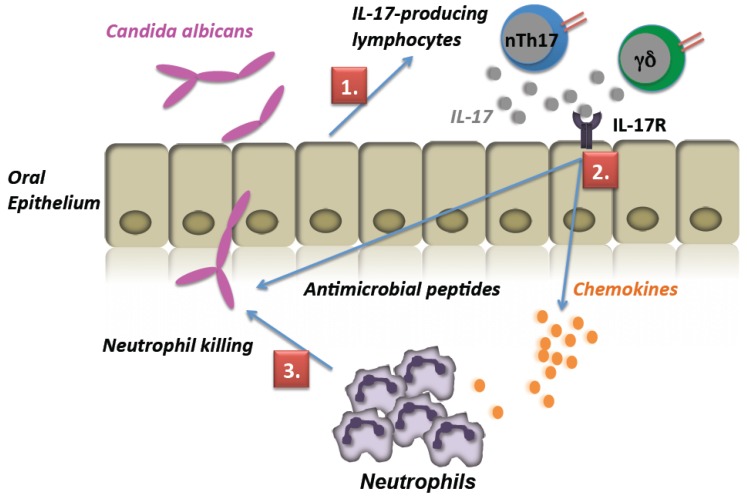
Sequence of IL-17-related immune activation events during oral *C. albicans* infection. 1, Upon exposure to *C. albicans* hyphae in the oral mucosa, oral mucosal cells induce factors that serve to activate and/or recruit IL-17A-producing lymphocytes, including γδ-T cells and natural Th17 cells [19,34]; 2, IL-17A produced by these cells interacts with its receptor, IL-17RA/IL-17RC, on the oral epithelial cells to activate a signaling program leading to characteristic gene expression. Genes induced by IL-17A include CXC chemokines, antimicrobial peptides and other immunoregulatory cytokines and transcription factors that participate in the response to OPC; 3, the induction of IL-17-dependent gene products contributes to neutrophil mobilization, candidacidal activity and control of fungal invasion [35].

IL-17 triggers the expression of a characteristic gene signature, including transcripts encoding chemokines and various antimicrobial genes [11,30]. *Cxcl5* and *Lcn2* are prototypical IL-17-induced genes that are expressed by oral epithelial cells following *C. albicans* infection. Consistent with the induction of IL-17A following infection, both of these genes were induced in isotype-treated mice after infection. In addition, a similar augmentation of gene expression was observed in neutrophil-depleted mice compared to isotype-treated mice (Figure 2B,C). The *Defb3* gene (encoding β-defensin 3) is another IL-17 target gene that is induced in epithelial cells upon IL-17 treatment [31,32,33] and is expressed constitutively in neutrophils; hence, its expression is markedly upregulated in the oral cavity following *Candida* infection. Indeed, *Defb3* was induced in infected WT mice treated with isotype Abs, as we have previously demonstrated [4]. Interestingly, *Defb3* was decreased in the neutrophil-depleted cohort compared to the isotype-treated control group (Figure 2D), which suggests that a significant portion of BD3 in OPC may come from the infiltrating neutrophils. Thus, IL-17A and epithelial-derived IL-17-dependent genes expressed by the oral epithelium, such as *Cxcl5* and *Lcn2*, are not dependent on neutrophils for expression. Rather, these findings support a model in which IL-17 is produced exclusively by TCR-bearing cell types, which induce the expression of neutrophil-recruiting genes and antimicrobial peptides, ultimately leading to *C. albicans* clearance (Figure 3).

## 3. Discussion

Neutrophils are the major innate immune cell recruited to sites of infection in response to extracellular pathogens. Although their major activities are typically considered phagocytic and microbicidal, neutrophils also express inflammatory and immunoregulatory cytokines and can thereby influence the overall immune response [36].

IL-17A and neutrophils are tightly interconnected. IL-17A signaling induces gene products that control neutrophils at the level of bone marrow activation (*i.e.*, G-CSF) or recruitment to sites of inflammation (e.g., CXCL1, CXCL5, IL-8) [37]. Neutrophil deficiency, arising from antibody-mediated depletion or defective recruitment, results in susceptibility to IL-17A-associated infections, such as OPC and periodontal disease [5,29,38]. Since neutrophils are infrequent in the oral mucosa prior to infectious challenge, the kinetics of neutrophil recruitment during oral *C. albicans* infection suggested that neutrophils are a downstream effector of IL-17A signaling. Indeed, IL-17RA^−/−^ and IL-17RC^−/−^ mice show reduced neutrophil recruitment to oral tissue following OPC [4,13]. Surprisingly, a recent report disputed the idea that IL-17A signaling is upstream of PMN recruitment, but to date, the basis for this discrepancy is not known [39]. In addition to acting upstream of IL-17A, several prior studies indicated that fungal and/or oral infections are associated with IL-17A production in neutrophils [22,23,24,25,40], and therefore, we sought direct evidence to determine whether neutrophils expressed IL-17A in response to *Candida* in the oropharynx.

Our results indicate that neutrophils recruited to the oral mucosa following *C. albicans* infection do not appear to produce IL-17A. Using IL-17A fate-tracking mice, we observed a reproducible, albeit small, subset of CD45^+^TCR^+^ cells in the tongue that express the YFP reporter gene, indicative of IL-17A expression at the transcriptional level (Figure 1A,B [19]). Although a previous report suggested that innate lymphoid cells (ILCs) produce IL-17 in this context [26], we found no evidence to support this conclusion, as all of the YFP^+^ cells expressed a TCR. In contrast, the Ly6G^+^ neutrophil population was not positive for YFP and, thus, did not produce IL-17A, either currently or any time in the past. Since neutrophils are short-lived, it is also unlikely that they simply produce IL-17 at a later time point, though we have not formally ruled this out. One caveat of the IL17^eYFP^ reporter mice is that there is a baseline threshold level of CRE expression necessary to generate detectable YFP in this system, and thus, it is possible to see IL-17A expression by intracellular cytokine staining (ICS), even in YFP-negative cells [20]. Thus far, we have not detected IL-17A expression by ICS in tongue homogenates, even in lymphocytes, probably due to the harsh experimental conditions needed to retrieve these cells from the collagen-rich environment of the tongue. Accordingly, we cannot formally rule out the possibility that low levels of IL-17 are expressed in non-lymphoid cell types, such as neutrophils.

The finding that *Il17a* mRNA levels are higher in mice treated with anti-Gr-1 Abs (Figure 2) supports the conclusion that neutrophils are not a vital source of this cytokine. Rather, neutrophil mobilization seems to negatively regulate IL-17 expression, a phenomenon that was previously shown to occur via control of IL-23 expression upon phagocytosis of apoptotic neutrophils [41]. This finding is also consistent with recent observations in periodontal disease associated with leukocyte adhesion deficiency [29]. However, it is possible that neutrophil depletion drives a process whereby other cell types (such as γδ-T or nTh17 cells) over-compensate by producing excess IL-17, explaining our findings.

Data in the literature both support and refute a role for neutrophil-derived IL-17A. Most notable for fungal immunology are the findings that *Aspergillus* and *Fusarium* exposure increases the frequency of neutrophils exhibiting IL-17A expression [22,23,24,25]. One report of IL-17^+^ neutrophils used extremely high dose cytokine treatment (in the microgram range) to prime neutrophils *in vitro* [25]. Nonetheless, it is possible that such elevated local concentrations of cytokines are found in certain settings and could consequently drive neutrophil IL-17 expression in some, but not all situations. For example, high concentrations of IL-6, IL-22 and IL-23 were observed in IL-17^+^ neutrophils in bullous pemphigoid blister fluid [42]. Insufficient concentrations of priming cytokines in the oral cavity might thus preclude the induction of IL-17^+^ neutrophils secondary to oral *Candida* infection*.* In a mouse arthritis model, neutrophils were reported to produce IL-17 in response to zymosan, an extract of yeast cell wall components [43], again suggesting a connection of fungal activation and neutrophilic IL-17 expression. IL-17 was also suggested to be produced by neutrophils in a mouse model of kidney ischemia reperfusion injury [44]. Conversely, neutrophils did not appear to express IL-17 in synovial biopsy tissue from arthritis patients [45]. Mast cells and neutrophils have also been suggested to express IL-17 in mouse models of psoriasis and spondyloarthritis [46,47,48,49]. It is unclear why neutrophils might express IL-17 in some tissues, but not others. One speculation is that immune privileged or sterile sites, such as the eye or kidney, do not have resident innate lymphocytes poised to produce this cytokine, whereas mucosal surfaces, such as the gut or mouth, are enriched in such cells.

Despite the many examples suggesting that IL-17 can be produced by non-lymphoid cells, proving cytokine production in neutrophils can be fraught with technical challenges, elegantly outlined in a recent review by Tecchio *et al.* [36]. In particular, neutrophils typically contain 10–20-fold less mRNA on a per-cell basis than other white blood cells, and therefore, even small numbers of contaminating cells can lead to false positive results. This may explain why it is common for researchers to isolate IL-17A mRNA in neutrophils, but harder to find convincing evidence for protein expression [23]. Another area in which data may be misleading is that IL-17 can be acquired externally via receptor-mediated internalization rather than translated *de novo*. Evidence for localization of IL-17A within neutrophils or other non-lymphoid cell types by immunohistochemistry or immunofluorescent staining may reflect this scenario. As noted above, mast cells were reported to be a source of IL-17 in the context of spondyloarthritis [46,47]. However, recent data support a model of IL-17A uptake through ligand-mediated internalization, allowing mast cells to store this cytokine for release at a later time [50,51]. Neutrophils likewise express high levels of IL-17RA that could serve to internalize IL-17A [52], which could lead to a positive signal in immunohistochemistry or immunofluorescent assays without actual intracellular production.

In summary, the role of PMNs as a source of IL-17A remains controversial. The data presented here provide evidence in a reporter mouse system that IL-17A is not expressed *de novo* in neutrophils in the context of acute oral candidiasis, despite a strong neutrophilic response. Rather, IL-17 is produced at very early stages by lymphocytes. This cytokine then acts upon responding cells within the oral mucosa to induce neutrophil-recruiting chemokines and antimicrobial peptides, and neutrophils subsequently facilitate the clearance of *Candida* organisms (Figure 3).

## 4. Experimental Methods

### 4.1. Mice and Oral Candidiasis Model

IL17^CRE^ mice, created by Brigitta Stockinger [20], were crossed to Rosa26^eYFP^ (The Jackson Laboratory, Bar Harbor, ME). All mice were on the C57BL/6 background, and all experiments included age- and sex-matched controls. OPC was performed by sublingual inoculation with a pre-weighed cotton ball saturated in 2 × 10^7^ cells/mL *C. albicans* (strain CAF2-1) or PBS (Sham) for 75 min under anesthesia, as described [4,53]. Oral swabs were obtained prior to each experiment to verify the absence of commensal *C. albicans* colonization. Mice were weighed daily and euthanized for humane reasons if they had more than a 25% weight loss or exhibited other signs of pain or distress (however, in these experiments, no animals fell into this category). There were no severe adverse events in any group. The animal protocols used in this work were evaluated and approved by the University of Pittsburgh Institutional Animal Care and Use Committee (Protocol 14125154, Animal Welfare Assurance Number A3187-01) and adhered to the guidelines in the Guide for the Care and Use of Laboratory Animals of the NIH.

### 4.2. Antibody-Mediated Neutrophil Depletion

Mice were injected intraperitoneally (i.p.) with Abs 24 h prior to inoculation with *C. albicans* (Day 0). Anti-Gr-1 (clone RB6-8C5, Bio X Cell, West Lebanon, NH, USA) and isotype control (clone LTF-2) Abs were administered at 80 μg/mouse [5]). Controls were injected with PBS.

### 4.3. Flow Cytometry

Flow cytometry of tongue tissue was performed as described [5]. Briefly, pooled tongues (*n =* 2–6 per sample) were processed with an enzyme cocktail (EDTA, HEPES, collagenase-2 (Worthington Biochemical, Lakewood, NJ, USA), dispase (Gibco, Invitrogen, Waltham, MA, USA), DNase I (Applied Biochemical) and defined trypsin inhibitor (Gibco)) and incubated at 37 °C for 45 min. Tissue was homogenized on a Miltenyi GentleMACS (Miltenyi Biotec Inc., San Diego, CA, USA) and passed through a cell strainer to form a single-cell suspension. The lymphocyte gate was verified by CD45 staining. Antibodies were from BD Biosciences (San Jose, CA, USA), eBioscience (San Diego, CA, USA) or Biolegend (San Diego, CA, USA). Samples (2 × 10^6^ cells each) were stained for CD11b-APC or PerCP-Cy5.5, Gr-1-Alexa Fluor 700 (AF700), CD45-AF700 or v450, Ly-6G-PE, CD44-E450, CD4-PerCP, TCRβ-PE, TCR-γδ-APC and Ly-6C-PerCP-Cy5.5. Matching isotype control antibodies and unstained specimens were used to establish gates. Data were acquired on an LSRII or LSR Fortessa (BD Biosciences, San Jose, CA, USA) and analyzed with FlowJo (Tree Star, Inc., Ashland, OR, USA).

### 4.4. RNA and qPCR

Frozen tongue was homogenized in RLT lysis buffer (RNAeasy Kit, Qiagen, Valencia, CA, USA) with a GentleMACS Dissociator (M-tubes, Miltenyi Biotec). RNA was extracted with the RNAeasy Kit. Approximately 0.5 μg total RNA were used to synthesize cDNA with a SuperScript III First-Strand Synthesis System (Invitrogen) and random hexamer primers. Quantification was determined by real-time PCR with SYBR Green (Quanta BioSciences, Gaithersburg, MD, USA) normalized to *Gapdh*. Primers were from SA Biosciences (Qiagen). Results were analyzed on a 7300 Real Time PCR System (Applied Biosystems, Carlsbad, CA, USA).

### 4.5. Statistics

At least two biological replicates were performed for all experiments. Data were compared by Mann–Whitney, ANOVA or unpaired Student’s *t*-test using GraphPad Prism (La Jolla, CA, USA) or Microsoft Excel software. *p*-values < 0.05 were considered significant.

## 5. Conclusions

Several studies have suggested that neutrophils may serve as sources of IL-17 during fungal infections, and IL-17 is vital for immunity to oropharyngeal candidiasis. We used a sensitive IL-17A-GFP reporter mouse system to show that this cytokine is not expressed by neutrophils in the immediate response to oral infection by *Candida albicans*.

## Abbreviations

OPC: oropharyngeal candidiasis
TCR: T cell receptor
CMC: chronic mucocutaneous candidiasis
YFP: yellow fluorescent protein
BD3: β-defensin 3
Ab: antibody
ICS: intracellular staining

## References

[1] Glocker E., Grimbacher B. (2010). Chronic mucocutaneous candidiasis and congenital susceptibility to Candida. Curr. Opin. Allergy Clin. Immunol..

[2] Milner J.D., Brenchley J.M., Laurence A., Freeman A.F., Hill B.J., Elias K.M., Kanno Y., Spalding C., Elloumi H.Z., Paulson M.L. (2008). Impaired T(h)17 cell differentiation in subjects with autosomal dominant hyper-IgE syndrome. Nature.

[3] Huppler A.R., Bishu S., Gaffen S.L. (2012). Mucocutaneous candidiasis: The IL-17 pathway and implications for targeted immunotherapy. Arthritis Res. Ther..

[4] Conti H., Shen F., Nayyar N., Stocum E., Sun J.N., Lindemann M., Ho A., Hai J., Yu J., Jung J. (2009). Th17 cells and IL-17 receptor signaling are essential for mucosal host defense against oral candidiasis. J. Exp. Med..

[5] Huppler A.R., Conti H.R., Hernandez-Santos N., Biswas P.S., Darville T., Gaffen S.L. (2014). Role of neutrophils in IL-17-dependent immunity to mucosal candidiasis. J. Immunol..

[6] Fidel P.L. (2011). Candida-host interactions in HIV disease: Implications for oropharyngeal candidiasis. Adv. Dent. Res..

[7] de Repentigny L., Lewandowski D., Joliceur P. (2004). Immunopathogenesis of oropharyngeal candidiasis in human immunodeficiency virus infection. Clin. Microbiol. Rev..

[8] Goupil M., Cousineau-Cote V., Aumont F., Senechal S., Gaboury L., Hanna Z., Jolicoeur P., de Repentigny L. (2014). Defective IL-17- and IL-22-dependent mucosal host response to *Candida albicans* determines susceptibility to oral candidiasis in mice expressing the HIV-1 transgene. BMC Immunol..

[9] Acosta-Rodriguez E.V., Rivino L., Geginat J., Jarrossay D., Gattorno M., Lanzavecchia A., Sallusto F., Napolitani G. (2007). Surface phenotype and antigenic specificity of human interleukin 17-producing T helper memory cells. Nature Immunol..

[10] Bär E., Gladiator A., Bastidas S., Roschitzki B., Acha-Orbea H., Oxenius A., LeibundGut-Landmann S. (2012). A novel Th cell epitope of *Candida albicans* mediates protection from fungal infection. J. Immunol.

[11] Hernández-Santos N., Huppler A.R., Peterson A.C., Khader S.A., McKenna K.C., Gaffen S.L. (2013). Th17 cells confer long term adaptive immunity to oral mucosal *Candida albicans* infections. Mucosal Immunol..

[12] Bishu S., Hernandez-Santos N., Simpson-Abelson M., Huppler A.R., Conti H.R., Ghilardi N., Mamo A., Gaffen S.L. (2014). CARD9 is required for adaptive but not innate immunity to oral mucosal *Candida albicans* infections. Infect. Immun..

[13] Ho A., Shen F., Conti H., Patel N., Childs E., Peterson A., Hernandez-Santos N., Kolls J., Kane L., Ouyang W. (2010). IL-17RC is required for immune signaling via an extended SEFIR domain in the cytoplasmic tail. J. Immunol..

[14] Puel A., Cypowji S., Bustamante J., Wright J., Liu L., Lim H., Migaud M., Israel L., Chrabieh M., Audry M. (2011). Chronic mucocutaneous candidiasis in humans with inborn errors of interleukin-17 immunity. Science.

[15] Ferreira M.C., Whibley N., Mamo A.J., Siebenlist U., Chan Y.R., Gaffen S.L. (2014). Interleukin-17-induced protein Lipocalin 2 is dispensable for immunity to oral candidiasis. Infect. Immun..

[16] Boisson B., Wang C., Pedergnana V., Wu L., Cypowyj S., Rybojad M., Belkadi A., Picard C., Abel L., Fieschi C. (2013). A biallelic Act1 mutation selectively abolishes interleukin-17 responses in humans with chronic mucocutaneous candidiasis. Immunity.

[17] Iliev I.D., Funari V.A., Taylor K.D., Nguyen Q., Reyes C.N., Strom S.P., Brown J., Becker C.A., Fleshner P.R., Dubinsky M. (2012). Interactions between commensal fungi and the C-type lectin receptor dectin-1 influence colitis. Science.

[18] Cua D.J., Tato C.M. (2010). Innate IL-17-producing cells: The sentinels of the immune system. Nat. Rev. Immunol..

[19] Conti H., Peterson A., Huppler A., Brane L., Hernández-Santos N., Whibley N., Garg A., Simpson-Abelson M., Gibson G., Mamo A. (2014). Oral-resident ‘natural’ Th17 cells and γδ-T cells control opportunistic *Candida albicans* infections. J. Exp. Med..

[20] Hirota K., Duarte J.H., Veldhoen M., Hornsby E., Li Y., Cua D.J., Ahlfors H., Wilhelm C., Tolaini M., Menzel U. (2011). Fate mapping of IL-17-producing T cells in inflammatory responses. Nature Immunol..

[21] Kagami S., Rizzo H.L., Kurtz S.E., Miller L.S., Blauvelt A. (2010). IL-23 and IL-17A, but not IL-12 and IL-22, are required for optimal skin host defense against *Candida albicans*. J. Immunol..

[22] Karthikeyan R.S., Vareechon C., Prajna N.V., Dharmalingam K., Pearlman E., Lalitha P. (2015). Interleukin 17 expression in peripheral blood neutrophils from fungal keratitis patients and healthy cohorts in southern India. J. Infect. Dis..

[23] Werner J.L., Gessner M.A., Lilly L.M., Nelson M.P., Metz A.E., Horn D., Dunaway C.W., Deshane J., Chaplin D.D., Weaver C.T. (2011). Neutrophils produce interleukin 17A (IL-17A) in a dectin-1- and IL-23-dependent manner during invasive fungal infection. Infect. Immun..

[24] Taylor P.R., Leal S.M., Sun Y., Pearlman E. (2014). Aspergillus and fusarium corneal infections are regulated by Th17 cells and IL-17-producing neutrophils. J. Immunol..

[25] Taylor P.R., Roy S., Leal S.M., Sun Y., Howell S.J., Cobb B.A., Li X., Pearlman E. (2014). Activation of neutrophils by autocrine IL-17A-IL-17RC interactions during fungal infection is regulated by IL-6, IL-23, RORγt and dectin-2. Nature Immunol..

[26] Gladiator A., Wangler N., Trautwein-Weidner K., Leibundgut-Landmann S. (2013). Cutting edge: IL-17-secreting innate lymphoid cells are essential for host defense against fungal infection. J. Immunol..

[27] Solis N.V., Filler S.G. (2012). Mouse model of oropharyngeal candidiasis. Nat. Protoc..

[28] Mehrad B., Strieter R.M., Moore T.A., Tsai W.C., Lira S.A., Standiford T.J. (1999). CXC chemokine receptor-2 ligands are necessary components of neutrophil-mediated host defense in invasive pulmonary aspergillosis. J. Immunol..

[29] Moutsopoulos N.M., Konkel J., Sarmadi M., Eskan M.A., Wild T., Dutzan N., Abusleme L., Zenobia C., Hosur K.B., Abe T. (2014). Defective neutrophil recruitment in leukocyte adhesion deficiency type I disease causes local IL-17-driven inflammatory bone loss. Sci. Transl. Med..

[30] Hernández-Santos N., Gaffen S.L. (2012). Th17 cells in immunity to *Candida albicans*. Cell. Host Microbe.

[31] Kao C.Y., Chen Y., Thai P., Wachi S., Huang F., Kim C., Harper R.W., Wu R. (2004). IL-17 markedly up-regulates beta-defensin-2 expression in human airway epithelium via JAK and NF-κB signaling pathways. J. Immunol..

[32] Kao C.Y., Kim C., Huang F., Wu R. (2008). Requirements for two proximal NF-κB binding sites and IκBζ in IL-17A-induced human beta-defensin 2 expression by conducting airway epithelium. J. Biol. Chem..

[33] Liang S.C., Tan X.Y., Luxenberg D.P., Karim R., Dunussi-Joannopoulos K., Collins M., Fouser L.A. (2006). Interleukin (IL)-22 and IL-17 are coexpressed by Th17 cells and cooperatively enhance expression of antimicrobial peptides. J. Exp. Med..

[34] Naglik J., Moyes D. (2011). Epithelial cell innate response to *Candida albicans*. Adv. Dent. Res..

[35] Conti H.R., Gaffen S.L. (2010). Host responses to *Candida albicans*: Th17 cells and mucosal candidiasis. Microbes Infect..

[36] Tecchio C., Micheletti A., Cassatella M.A. (2014). Neutrophil-derived cytokines: Facts beyond expression. Front. Immunol..

[37] Iwakura Y., Ishigame H., Saijo S., Nakae S. (2011). Functional specialization of interleukin-17 family members. Immunity.

[38] Yu J., Ruddy M., Wong G., Sfintescu C., Baker P., Smith J., Evans R., Gaffen S. (2007). An essential role for IL-17 in preventing pathogen-initiated bone destruction: Recruitment of neutrophils to inflamed bone requires IL-17 receptor-dependent signals. Blood.

[39] Trautwein-Weidner K., Gladiator A., Nur S., Diethelm P., LeibundGut-Landmann S. (2015). IL-17-mediated antifungal defense in the oral mucosa is independent of neutrophils. Mucosal Immunol..

[40] Eskan M.A., Jotwani R., Abe T., Chmelar J., Lim J.H., Liang S., Ciero P.A., Krauss J.L., Li F., Rauner M. (2012). The leukocyte integrin antagonist Del-1 inhibits IL-17-mediated inflammatory bone loss. Nature Immunol..

[41] Stark M.A., Huo Y., Burcin T.L., Morris M.A., Olson T.S., Ley K. (2005). Phagocytosis of apoptotic neutrophils regulates granulopoiesis via IL-23 and IL-17. Immunity.

[42] Le Jan S., Plee J., Vallerand D., Dupont A., Delanez E., Durlach A., Jackson P.L., Blalock J.E., Bernard P., Antonicelli F. (2014). Innate immune cell-produced IL-17 sustains inflammation in bullous pemphigoid. J. Invest. Derm..

[43] Milanova V., Ivanovska N., Dimitrova P. (2014). TLR2 elicits IL-17-mediated RANKL expression, IL-17, and OPG production in neutrophils from arthritic mice. Mediators Inflamm..

[44] Li L., Huang L., Vergis A.L., Ye H., Bajwa A., Narayan V., Strieter R.M., Rosin D.L., Okusa M.D. (2010). IL-17 produced by neutrophils regulates IFN-γ-mediated neutrophil migration in mouse kidney ischemia-reperfusion injury. J. Clin. Invest..

[45] van Baarsen L.G., Lebre M.C., van der Coelen D., Aarrass S., Tang M.W., Ramwadhdoebe T.H., Gerlag D.M., Tak P.P. (2014). Heterogeneous expression pattern of interleukin 17A (IL-17A), IL-17F and their receptors in synovium of rheumatoid arthritis, psoriatic arthritis and osteoarthritis: Possible explanation for nonresponse to anti-IL-17 therapy?. Arthritis Res. Ther..

[46] Noordenbos T., Yeremenko N., Gofita I., van de Sande M., Tak P.P., Canete J.D., Baeten D. (2012). Interleukin-17-positive mast cells contribute to synovial inflammation in spondylarthritis. Arth. Rheum..

[47] Hueber A.J., Asquith D.L., Miller A.M., Reilly J., Kerr S., Leipe J., Melendez A.J., McInnes I.B. (2010). Mast cells express IL-17A in rheumatoid arthritis synovium. J. Immunol..

[48] Keijsers R.R., Joosten I., van Erp P.E., Koenen H.J., van de Kerkhof P.C. (2014). Cellular sources of IL-17 in psoriasis: A paradigm shift?. Exp. Derm..

[49] Lin A.M., Rubin C.J., Khandpur R., Wang J.Y., Riblett M., Yalavarthi S., Villanueva E.C., Shah P., Kaplan M.J., Bruce A.T. (2011). Mast cells and neutrophils release IL-17 through extracellular trap formation in psoriasis. J. Immunol..

[50] Noordenbos T., Paramarta J., Blijdorp I., Baeten D. (2014). Human mast cells engulf and store exogenous IL-17A. Clin. Exp. Rheumatol..

[51] Baeten D. (2015). Personal communication.

[52] Ishigame H., Kakuta S., Nagai T., Kadoki M., Nambu A., Komiyama Y., Fujikado N., Tanahashi Y., Akitsu A., Kotaki H. (2009). Differential roles of interleukin-17A and -17F in host defense against mucoepithelial bacterial infection and allergic responses. Immunity.

[53] Kamai Y., Kubota M., Kamai Y., Hosokawa T., Fukuoka T., Filler S. (2001). New model of oropharyngeal candidiasis in mice. Anti-microb. Agents Chemo..

